# Electroacupuncture for relieving itching in atopic eczema: study protocol for a multicenter, randomized, sham-controlled trial

**DOI:** 10.3389/fmed.2023.1320230

**Published:** 2023-12-20

**Authors:** Si-han Wang, Rui-long Liang, Han Yang, Xiao-ce Cai, Jiao Wang, Xiao-ying Sun, Jia-le Chen, Chun-xiao Wang, Wen-cheng Jiang, Xin Li

**Affiliations:** ^1^Department of Dermatology, Yueyang Hospital of Integrated Traditional Chinese and Western Medicine, Shanghai University of Traditional Chinese Medicine, Shanghai, China; ^2^Institute of Dermatology, Shanghai Academy of Traditional Chinese Medicine, Shanghai, China; ^3^Department of Acupuncture, Yueyang Hospital of Integrated Traditional Chinese and Western Medicine, Shanghai University of Traditional Chinese Medicine, Shanghai, China; ^4^Shanghai Skin Disease Hospital, School of Medicine, Tongji University, Shanghai, China

**Keywords:** electroacupuncture (EA), atopic eczema (AE), pruritus severity, randomized controlled trial, park sham device (PSD)

## Abstract

**Background:**

Atopic eczema (AE) is a common atopic inflammatory skin disease affecting 2.1–4.9% of the population in different countries. Pruritus, one of the most burdensome symptoms, is often underestimated for the problems it can cause, creating a vicious loop of itching, scratching, and lichenification. Therefore, further research into practical and safe treatments that relieve itchy symptoms and enhance skin protection is key to overcoming AE. Acupuncture, with or without electrical stimulation, is one of the most commonly used therapeutic measures to treat AE. This trial aimed to objectively evaluate the efficacy and safety of the electroacupuncture (EA) antipruritic technique in AE pruritus and obtain high-level clinical evidence for the popularization and application of EA for AE.

**Methods and analysis:**

This multicenter, single-blinded, randomized controlled trial is planned to transpire from April 15, 2023, to June 30, 2025. We will recruit 132 participants with AE (44 per group). Participants will be assigned randomly to three equal-sized groups: EA, sham electroacupuncture, and sham acupuncture. Treatment will be administered three times a week during the 2-week intervention phase. The primary outcome measure is the Visual Analog Scale, with a numeric rating scale to evaluate pruritus. Secondary outcome measures include the Eczema Area and Severity Index and Dermatology Life Quality Index. Other outcome measures include physical examination, serum IgE, and safety evaluation. The number, nature, and severity of adverse events will be carefully recorded.

**Trial registration:**

ClinicalTrials.gov, 22Y11922200. Registered 3 September 2022, https://register.clinicaltrials.gov.

## Introduction

Atopic eczema (AE) is a common inflammatory condition characterized by itchy, scaly, erythematous, and oozing skin. The prevalence of AE in adults ranges from 2.1 to 4.9% in different countries (1). Confusion and inconsistency in their usage have resulted from the concurrent use of words including “eczema,” “atopic eczema,” “atopic dermatitis,” and “atopic eczema/dermatitis syndrome” (2). Therefore, “atopic eczema” has been proposed as a unifying term by the World Allergy Organization’s nomenclature review committee and is used throughout the text of this protocol (3).

Clinically, AE is usually classified into acute, sub-acute, and chronic types according to the lesion’s appearance. Erythema, papules, vesicles, and serous effusion are signs of acute lesions (4). Chronic AE is characterized by symmetry, serous exudate, pruritus, and recurrence; its symptoms include intractable itching, lichenification, and hyperpigmentation (5).

Itching is a burdensome symptom of AE and can lead to scratching, which can increase lesion inflammation and cause secondary skin infections. Reducing scratching behavior reduces lesion inflammation and promotes skin healing (6, 7). Itch-scratching behaviors lead to sleep disturbance, sleep-related impairment, and even physical and psychological disorders (8, 9). Skin lesions from long-term scratching progress to lichenification, creating a vicious cycle of “itching, scratching, and lichenification” that triggers and exacerbates AE (10). AE’s relapsing and remitting nature makes controlling intractable itching a challenge, considerably burdens a patient’s cost of living, and seriously affects their quality of life (5). Therefore, controlling itching is an important objective of AD treatment.

AE is a complex atopic skin disease caused by multiple factors, including genetic, external, environmental, and other factors affecting pathogenesis. The most commonly used clinical treatments for pruritus associated with AE include topical corticosteroids (TCS) and oral H1-antihistamines.

TCSs are an effective first-line treatment for AE. However, dependency and TCS withdrawal (addiction) are common; prolonged and extensive TCS use can result in adverse skin and systemic effects (11). Consequently, patients tend to have negative feelings toward TCS (12). Since TCS phobia decreases patient compliance, it is a major factor in treatment failure in patients suffering from AE (13). Therefore, TCS may be insufficient for controlling pruritus in AE.

The *American Academy of Dermatology* (AAD), *the European guidelines for chronic pruritus*, and a position paper from *the International Society of Atopic Dermatitis (ISAD)/Oriented Patient-Education Network in Dermatology (OPENED)* advise against the general use of H1-antihistamines for managing AE (14–17). However, they remain commonly used in the clinic because of their clinical efficacy in sedating, relieving itching, and improving sleep disturbances (15). The older first-generation H1-antihistamines are discouraged for long-term use, especially in children, because of drowsiness and adverse effects on learning and cognitive functions. Although second-generation H1-antihistamines have fewer side effects and better efficacy, some cause drowsiness, and some have cardiotoxic effects. Moreover, both generations of H1-antihistamines burden liver and kidney functions (18, 19). Therefore, further research into practical and safe treatments that relieve itchy symptoms and enhance skin protection is important for overcoming AE.

Acupuncture has recently gained importance in dermatology as a common complementary and alternative medicine therapy (20). With or without electrical stimulation, acupuncture can reduce lesions, ease itching, and improve patient quality of life (21–24).

Based on an animal study, acupuncture is more effective than other histamine-independent pruritogens (chloroquine) or histamine-dependent pruritogens (compound 48/80). The powerful inhibitory effects of acupuncture on serotonergic itch are likely caused, at least in part, by inhibiting 5-HT2 and 5-HT7 receptors (25, 26). Electroacupuncture (EA) has been shown to alleviate itch and flare in healthy human adults after intradermal histamine injection (27–29). Animal studies have shown that EA relieves pruritus; the underlying anti-inflammatory effects are closely linked to dynorphin release (30). Later research suggested that the vagal–adrenal anti-inflammatory axis is likely mediated by PROKR2ADV sensory fibers that innervate the deep limb fascia by low-intensity electropuncture stimulation. These studies provide a neuroanatomical basis for the selectivity and specificity of acupoints in driving specific autonomic pathways (31).

High-quality randomized controlled trials remain lacking despite various clinical studies on acupuncture therapy for AE in the treatment of pruritus. This study proposes a design for a multicenter, randomized, sham electroacupuncture–controlled protocol aiming for an objective and standardized evaluation of the acupuncture antipruritic technique’s effect on pruritus, clinical efficacy, and safety in AE to obtain definitive clinical evidence. The ultimate goal is to develop a clinical protocol suitable for standardizing the widely used acupuncture antipruritic techniques for AE.

## Methods and analysis

### Study setting

This multicenter, single-blinded, randomized controlled trial will be conducted in the Yueyang Hospital of Integrated Traditional Chinese and Western Medicine and the Shanghai Skin Disease Hospital in China. The trial has been registered at ClinicalTrials.gov (ID: 22Y11922200). At the time of writing this report (version 2.0, January 4, 2023), the trial has not yet begun enrollment. Participant recruitment will take place from April 15, 2023, to June 30, 2025. This protocol has been developed according to Standard Protocol Items: Recommendations for Interventional Trials (SPIRIT) 2013. The checklist is presented in detail in Supplementary Table 1.

### Sample size

According to a previous study (32), the mean itch intensity was taken as 35.7% for the EA group, 40.4% for the sham electroacupuncture (SEA) group, and 45.9% for the sham acupuncture (SAC) group, with *α* = 0.05, test efficacy 1-β = 0.8, two-sided test, using the estimation of sample size formula for comparing more than two treatment groups [referring to $n=2{\left(\sigma \frac{Z_{1-\alpha /\left(2\tau \right)}+Z_{1-\beta }}{\mu_{A}-\mu_{B}}\right)}^{2}$ , $1-\beta =\Phi \left(Z-Z_{1-\alpha /\left(2\tau \right)}\right)+\Phi \left(-Z-Z_{1-\alpha /\left(2\tau \right)}\right)$ , $Z=\frac{\mu_{A}-\mu_{B}}{\sigma \sqrt{\frac{2}{n}}}$ , where Φ is the standard normal cumulative distribution function, $\mu_{A}\;\mathrm{and}\;\mu_{B}$ representing the mean of any two groups, and n is the sample size of a pair of τ comparisons (taking the maximum value)]. Consequently, the sample content was calculated to be 35 cases per group and 105 cases for the three groups. Assuming a 20% dropout rate of patients at follow-up, we calculated the sample size to be 44 cases for each group and 132 participants for the three groups (EA, SEA, and SAC). Participants will be recruited from two research centers, each targeting 66 participants.

### Eligibility criteria

Inclusion criteria:

To be eligible for recruitment, the patient must:

- Be diagnosed with AE for a duration longer than 2 months.
- Be aged 18–65 years (male or female).
- Have body surface area (BSA) scores <10%.
- Provide consent to participate in clinical trial observation and cooperation with scheduled follow-ups.
- Sign and date the informed consent form.

Exclusion criteria:

Patients who meet any of the following criteria will be excluded:

- Have been systematically treated with drugs, such as hormones or immunosuppressants, within 2 weeks.
- Do not tolerate acupuncture.
- Signs of serious secondary infections, including localized skin infections and infections of other organs.
- Pregnancy, lactation, or planning to become pregnant during the trial.
- Diabetes or a combination of severe heart, liver, or kidney diseases.
- Received other investigational drugs or participated in other clinical trials within 3 months.
- BSA scores ≥10%.
- Unable to cooperate with the study for any reason, such as impaired language comprehension or inability to visit the study center.
- Generalized eczema with lesions spreading over the body is unsuitable for acupuncture.
- Acute flare-ups.

Drop-out exclusion criteria:

Patients who meet any of the following criteria will be a dropout case; if the time is past the mid-point of participation in the overall study, the data should be included in the efficacy statistics:

- Experienced unexpected events during treatment and are unfit to continue with the trial.
- Develop other illnesses during the course of the trial that affect judgments of efficacy and safety.
- Lack of compliance or voluntarily accepting other treatments during the course.
- Information is incomplete and affects judgments of efficacy and safety.
- Changed their contact details, resulting in loss of contact.

Exit/termination criteria:

- Patients who have experienced severe adverse reactions during treatment and whose treating physician assessed that the trial is no longer appropriate.
- Patients’ unsolicited requests to withdraw from the trial should be recorded, and the reasons noted.

### Randomization and allocation

Once eligibility is confirmed and consent is obtained, participants will be block-randomized using a non-stratified, permuted block of varying lengths (*n* = 2 blocks; patient distribution = 1:1:1) for each group. Independent statisticians will provide computer-generated random sequences. Distribution will be conducted using a central web-based interactive randomization service system; the patients will be assigned randomly to three groups. Participant identification numbers will be provided to make identification easier throughout the trial. The researchers who perform random grouping will need to log onto the website to view the grouping situation of each eligible participant. The authors have no access to information that could identify individual participants during or after data collection.

### Blinding

Because of acupuncture’s nature, blinding the physician involved in the treatment application is impossible. Each hospital will designate several operating researchers who are dermatologists trained to perform EA therapy and obtain relevant operational qualifications. Therefore, operating researchers will not participate in the statistical analysis; the treatment plan and grouping will be known only by statistical analysts. To ensure blinding, we will choose the Park Sham Device (PSD) as a placebo control in a single-blind clinical trial, use a way of not energizing the EA device, and use black eye masks to shield the participants’ eyes. The treatment device will also be the same size and material, and the treatment method will maintain a consistent frequency and intensity.

In the case of serious adverse events, the principal investigators at each participating center will decide whether allocation concealment needs to be broken. If allocation concealment is broken before the end of the trial, the data will be censored from analysis and the patients will be considered to have dropped out of the trial.

### Study timeline

**Figure 1:**
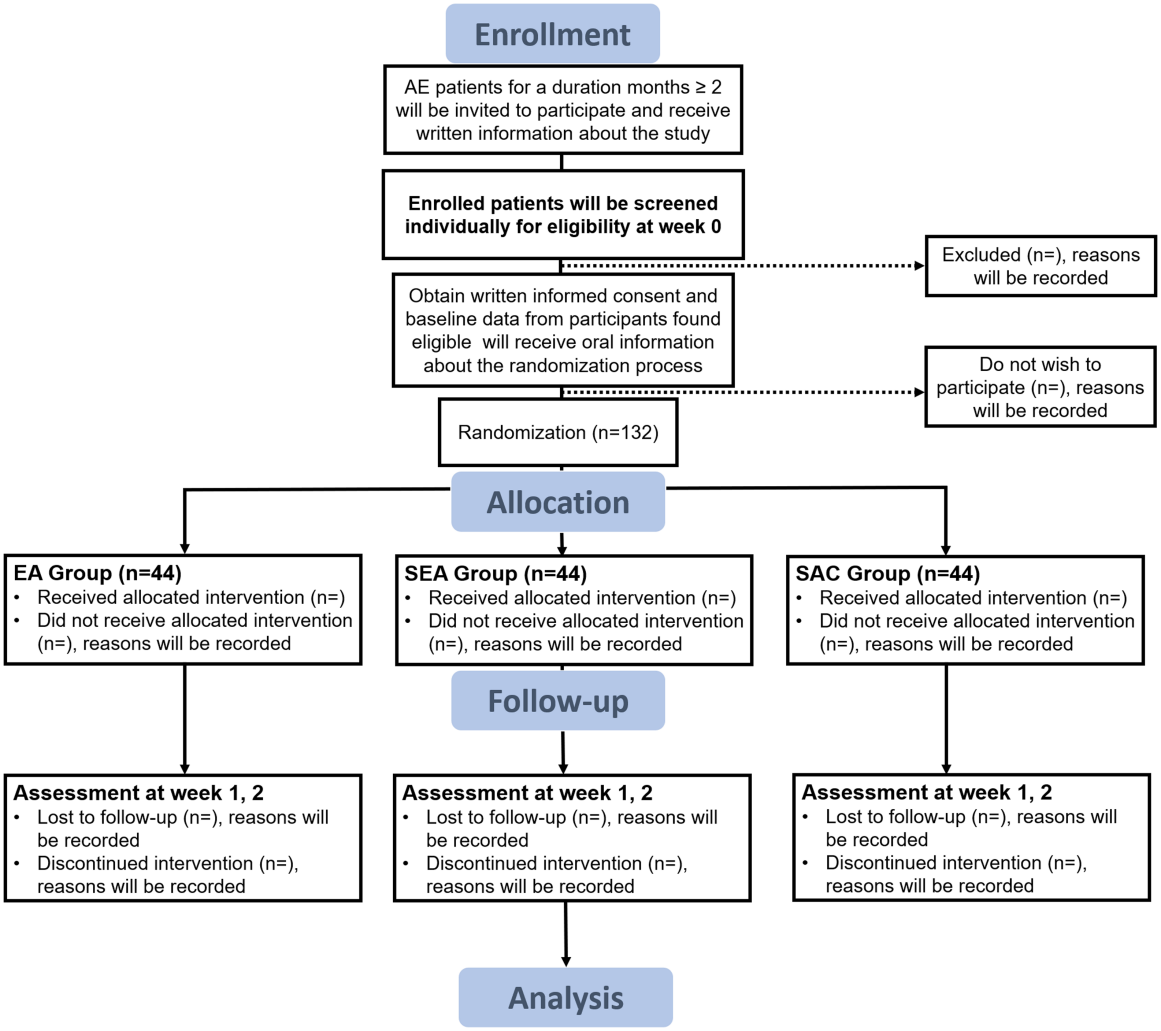
Flow diagram. AE, atopic eczema; EA, electroacupuncture; SEA, sham electroacupuncture; SAC, sham acupuncture.

The study protocol consists of two main phases over 3 weeks: the screening and the treatment phase (Figure 1). The screening period will last 1 week; participants will be included in this phase according to the inclusion/exclusion criteria. Participants will be treated three times a week for 2 weeks, with different interventions among the groups.

### Screening phase

Patients with AE will be enrolled in the screening phase through a dermatological outpatient service, and physical examinations and inclusion evaluations will be performed. The trial’s eligible participants will be requested to complete a written informed consent form (including the procedures, risks, and options for quitting the study). The study will be thoroughly explained to the participants by a researcher with the required training. Participant must sign an informed consent form after giving their approval. An algorithm will separate patients randomly into the EA, SEA, and SAC groups.

### Treatment phase

**Figure 2:**
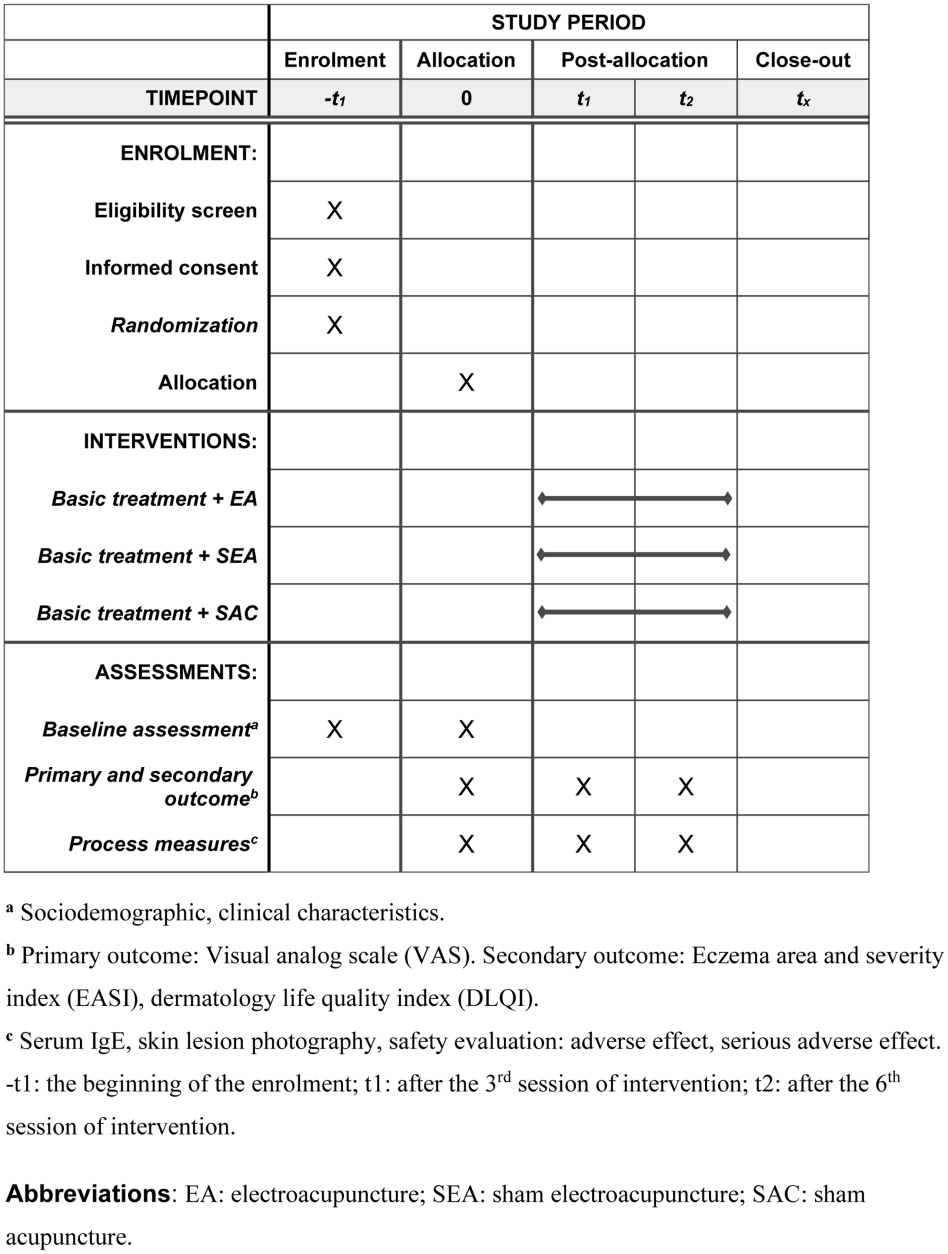
Schedule of enrollment, interventions, and assessments.

Patients with AE will receive interventions during the treatment phase. Patients will be checked for visual analog scale (VAS), Eczema Area and Severity Index (EASI), Dermatology Life Quality Index (DLQI), serum IgE, adverse events, and compliance during the two-week therapy period, administered three times a week. After 2 weeks, all interventions will end. We will record demographic characteristics, medical and medication histories, images of skin lesions, personal life histories, vital signs, physical examinations, and adverse effects (among other data) (Figure 2).

### The park sham device (PSD)

**Figure 3:**
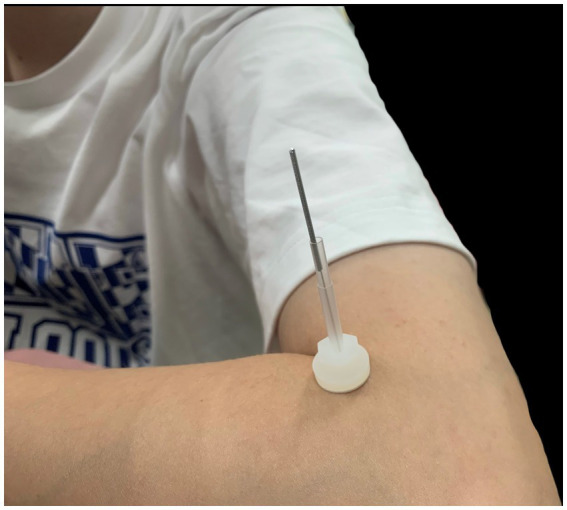
The park sham device attached to the skin.

The PSD and Park sham needle (PSN) are purchased from AcuPrime.^1^ We chose the PSD to maximize the blinding of participants because it has a double-tubed system that attaches to a plastic base and the participant’s skin (Figure 3). Double-sided tape is used to secure the device to the plastic base and the participant’s skin. Initially, in its expanded (highest) position, the inner tube is lowered to the outer tube as the needle is lowered. A real acupuncture needle or a PSN can be stored inside the PSD. The PSN is stainless steel with a length measuring 25–50 mm and looks like and is packaged similarly to an acupuncture needle. When downward pressure is applied to the handle of the PSN, the needle glides into the handle, providing the illusion that it is piercing the skin. PSD is critical to prevent the PSN from falling away from the participant’s skin and keep them from noticing whether a PSN or an actual acupuncture needle is used.

### Intervention

All three groups will have the PSD placed by the researchers. It will be applied to standard acupuncture points in the EA and SEA groups and sham acupuncture points in the SAC group.

**Figure 4:**
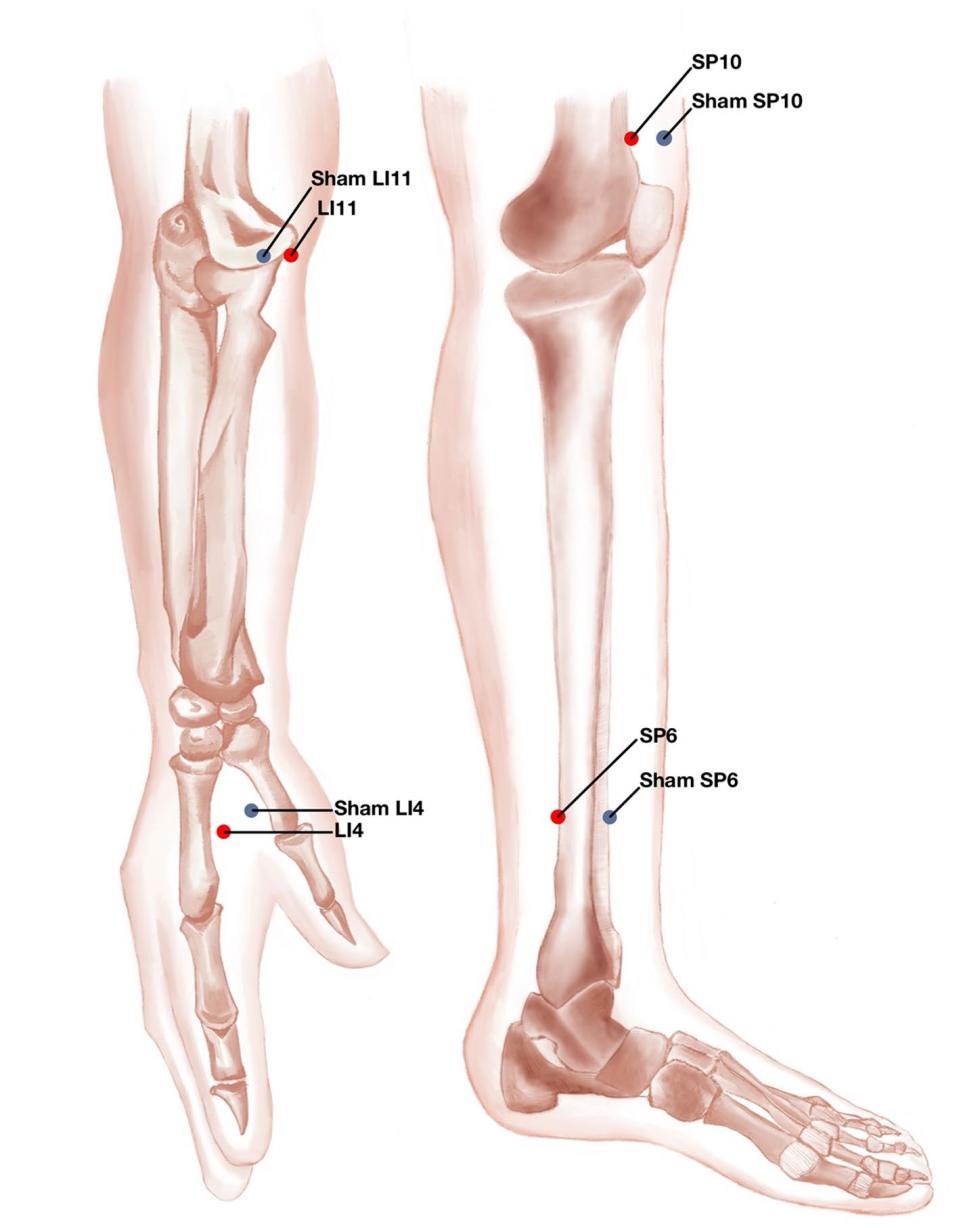
Location of verum acupoints and sham acupoints.

Participants in the EA group will receive acupuncture with PSD at the bilateral *Hegu* (LI4, located between the first and second metacarpals, at the midpoint of the radial aspect of the second metacarpal), bilateral *Quchi* (LI11, located at the lateral end of the transverse elbow stripe, flexing the elbow, at the midpoint of the line connecting the *Chize* (LU5) and the external humeral epicondyle), bilateral *Xuehai* (SP10, located on the medial thigh, flexing the knee, 2 cun [≈10 mm] above the medial end of the base of the patella, at the bulge of the medial head of the quadriceps), and bilateral *San Yinjiao* (SP6, located 3 cun [≈ 10 mm] above the tip of the inner ankle, on the posterior edge of the medial side of the tibia) (Figure 4). After skin disinfection, the acupuncture needles are inserted approximately 10–20 mm into the skin at a 90° angle.

Achieving qi (a combination of sensations, including soreness, numbness, distention, heaviness, and other sensations) is considered an important part of obtaining the efficacy of acupuncture. Four pairs of electrodes are used: two each on bilateral *Quchi* (LI4) and *Hegu* (LI11) and two each on bilateral *Xuehai* (SP10) and *San Yinjiao* (SP6); they are all ipsilateral to one another. The duration of the EA stimulation is 30 min, with the current intensity of 1–5 mA with a continuous wave of 20 Hz (with the skin around the acupoints shivering mildly without pain). For 2 weeks straight, the participants will have three therapy sessions each week for a total of six sessions.

Participants in the SEA group will receive SEA with PSD but without energizing the electropuncture device. Procedures, electrode placements, and other treatment settings will be the same as those in the EA group but with no electricity output.

Participants in the SAC group will receive sham acupuncture with PSD and PSN at sham acupoints (non-meridian and non-acupuncture points). The sham acupoints selected for this study, based on reports from other scholars, are as follows: (33) The sham *Hegu* (LI11) is located at the dorsum of the hand, between the first and second metacarpals, 1 cun [≈10 mm] next to the midpoint of the radial side of the second metacarpal. The sham *Quchi* (LI4) point is at the midpoint between the locations of verum *Quchi* and *Chize*; the sham *Xuehai* (SP10) point is 1 cun [≈10 mm] lateral to the verum *Xuehai*; the sham *San Yinjiao* (SP6) point is at 3 cun [≈10 mm] above the tip of the lateral ankle, between the stomach and bile meridians.

Procedures, electrode placements, and other treatment settings will be the same as those in the EA group, but with no skin penetration, electricity output, or needle manipulation to achieve qi.

#### Basic treatment

Throughout the course of the trial, all patients will receive standard base treatment (health guidelines).

1. Increase moisturizing of the skin: As a basic treatment, using a moisturizer is mandatory (34). The moisturizer should be applied to skin areas free from erosion, exudation, and dry non-lesion areas. A soft, fragrance-free moisturizer will be used in this study. Urea cream (purchased from Yueyang Hospital of Integrated Traditional Chinese and Western Medicine, H04050489) is recommended for patients with sensitive, dry skin.
2. Standard bath: Generally, patients should rinse quickly for 5 min once daily in warm water (35–39°C). To prevent the skin from becoming dehydrated after showering, a moisturizer should be applied within 2 min. We recommend avoiding cleaning the skin with alkaline detergents.
3. Avoiding factors that cause or worsen disease: Some diets might cause or aggravate disease for certain people. Avoiding these foods is advised.
4. Some patients may have food allergies. When food allergies are discovered, certain foods should be avoided to avoid causing or exacerbating the disease.
5. Maintain a moderately healthy lifestyle: patients should abstain from excessive stress and staying up late. Spicy and irritating foods should be avoided, and appropriate exercise should be performed. Patients should attempt to maintain regular bowel movements.

### Drug combination

#### Combination treatments permitted

Patients with co-morbidities or newly developed co-morbidities will be allowed to take specific medications/other treatments during the study, but only if the details are included in the medical record for analysis. The drug name (or other therapeutic name), dosage, frequency, and time of use are recorded for convenient summary and analysis.

#### Combination treatment prohibited

1. Other herbal medicinal preparations for the purpose of treating the disease.
2. Other topical preparations containing drugs, including steroid preparations, coal tar, herbal preparations, etc.
3. Antihistamines.
4. Systemic steroid hormone therapy.
5. Non-steroidal immunosuppressive agents.
6. Photochemotherapy.
7. Desensitization therapy.

### Outcome measures

#### Primary outcome measure

The proposed study’s primary outcome is improved VAS scores after 2 weeks of treatment. VAS scores are designed to be measured at the screening phase, after 1 week of treatment, and after 2 weeks of treatment. Pruritus, the main clinical symptom of eczema, can be scored separately on the VAS (35).

#### Secondary outcome measure

Secondary outcome measures include the EASI (36) and DLQI (37). The timeline for the efficacy evaluation is shown in Figure 2.

#### Safety evaluation

Several measures will be taken for safety, including the observation of vital signs, drug combination, physical examination, adverse events, and severe adverse events outcomes. The timeline for the safety evaluation is presented in Figure 2. If an adverse event occurs, the researchers must quickly attend to it and record the date of occurrence, severity, duration, and treatment measures. In the case of a serious adverse event, the researchers must take immediate action to ensure the safety of the participants, report to the principal investigators and Western Medicine and the Ethics Committee within 24 h, and determine if the participant should be withdrawn based on the condition.

### Data management and monitoring

All study data, including informed consent forms, will be collected and maintained in a dedicated database for medical research records and must be submitted by researchers at the end of the treatment phase. The principal investigators at the participating hospitals will be responsible for collecting the medical research records from the researchers and for reviewing and storing these records.

The data report adopts an electronic Case Report Form (eCRF); each center will assign a specific person to data entry. To ensure the quality and consistency of the source data and database input, two researchers will independently verify the source data with the eCRF data. In the case of any discrepancy, a query list will be created, and the researchers must respond to the query list to resolve the discrepancy. All documentation related to quality control will be maintained to allow for future objective evaluation of safety and critical efficacy outcomes.

### Statistical analysis

Professional statisticians developed a statistical analysis plan upon consultation with the primary investigators. The data will be stored in the Data Management Center of Jiangsu Famous Medical Technology Co. Ltd. and processed by in-house statisticians blinded to group allocation.

All statistical analyses are performed using R [version 4.1.3; R Core Team (2022). R: A language and environment for statistical computing. R Foundation for Statistical Computing, Vienna, Austria^2^]. Statistical significance is set at value of *p* < 0.05. The intention-to-treat analysis population, consisting of all participants who pass the randomization stage, will be the primary population for demographic and other baseline characteristics, compliance, efficacy analysis, and safety analysis.

The analytical methods will be combined with descriptive and comparative analyses. Qualitative data will be described by frequency tables, percentages, or composition ratios; comparative analyses will be conducted using the chi-squared test, Fisher’s exact test, Wilcoxon rank-sum test, Cochran–Mantel–Haenszel test, and weighted least-squares covariance. Quantitative data will be described as the mean, standard deviation, median, quartile, minimum, and maximum. The *t*-test will be used for comparative analysis of data with normal distributions and with Satterthwaite correction if the variance is uneven. Quantitative data exhibiting a non-normal distribution on the Wilcoxon rank-sum test will be analyzed using the Wilcoxon signed-rank sum test and covariance generalized linear models. A sensitivity analysis for the primary outcome with the most conservative data (the worst or best outcome of that indicator in the group) will assess the robustness of the missing data at random assumption. A multiple imputation will be used to impute missing values.

The hypothesis test is two-sided; the test statistics and their corresponding *p*-values will be reported.

### Patients and public involvement

The patients and the public will not be involved in the design, conduct, reporting, or dissemination plans of our research.

## Discussion

AE and other allergies are becoming more common on globally (38). AE is a common, recurrent, and inflammatory skin condition. In China, 15–30% of dermatological outpatient cases have AE (39). One of the most common chronic conditions, AE, affects up to a fifth of the population in developed countries. It was once thought to be the initial symptom of atopy—the tendency for a family member to develop IgE sensitivity to environmental allergens—and the onset of “atopic march,” which ultimately leads to asthma and allergic rhinitis (40, 41). Quality of life and economic outcomes can be adversely affected, particularly for people with moderate to severe AE.

Itching is one of the most burdensome symptoms of AE; the problems caused by itching cannot be underestimated. Severe itching often leads to excessive scratching, which indirectly leads to lichenification, thus transitioning from acute to sub-acute eczema, and ultimately to relapsing AE. Therefore, one goal of AE treatment should be addressing the pruritic symptoms in patients.

AE has no curative therapy. The basic idea is to avoid certain triggers, continuously restore the epidermal barrier using emollients, and treat inflammation with TCSs or antihistamines. Because of the limited number of trials explicitly comparing medications, how well different treatments control the condition over time is unknown (42, 43).

Acupuncture, a complementary and alternative medicine approach, can help overcome these limitations, effectively relieve itching, and improve patients’ quality of life (44–46). EA decreases serum levels of neuropeptides and inflammatory factors, making it effective for allergic diseases, and has been increasingly utilized as an adjuvant therapy in routine eczema treatment. Numerous clinical studies on using acupuncture or EA for AE have been reported (47–52).

We propose a prospective, multicenter, randomized controlled study of AE. The multicenter model considers the effect of environmental factors and individual participant characteristics on trial results, providing more reliable clinical evidence. We designed three arms to compare the effectiveness of EA and acupuncture and to establish the genuineness and credibility of the acupoints while eliminating any potential placebo effect. The objective VAS score was adopted as the primary outcome measure, and the EASI and DLQI scores as the secondary outcome measures, which should improve the reliability of the results. In addition, physical examination, personal life history, safety evaluation, patient satisfaction, and other outcome measures have been added. Keeping participants closer to the clinic helps track the disease progression in a clinical setting.

The study design has limitations. First, the sample size is only 132 participants. Second, the study duration is limited to 3 weeks without a follow-up period. Observing the long-term effects of the EA anti-itch technique on the progression of AE with a limited sample size and follow-up period will be challenging. However, we will use unique experimental designs and outcome measures to improve the accuracy and sensitivity of this study to mitigate these limitations.

In summary, this multicenter, randomized, sham electroacupuncture–controlled study will assess the effect on pruritus, clinical efficacy, and safety of the acupuncture antipruritic technique in AE. We will also establish a multicenter clinical registration platform and evaluate clinical evidence by assessing the treatment’s therapeutic benefits (clinical effectiveness with no adverse side effects). The findings will aid decision-making for managing and treating AE and offer crucial data that could be integrated into future clinical recommendations.

## Data availability statement

The raw data supporting the conclusions of this article will be made available by the authors, without undue reservation.

## Ethics statement

The studies involving humans were approved by Ethics Committee of Yueyang Hospital of Integrated Traditional Chinese and Western Medicine. The studies were conducted in accordance with the local legislation and institutional requirements. The participants provided their written informed consent to participate in this study. Written informed consent was obtained from the individual(s) for the publication of any potentially identifiable images or data included in this article.

## Author contributions

S-hW: Data curation, Formal analysis, Investigation, Software, Visualization, Writing – original draft, Writing – review & editing. R-lL: Data curation, Formal analysis, Investigation, Project administration, Writing – original draft. HY: Data curation, Formal analysis, Investigation, Project administration, Writing – original draft. X-cC: Data curation, Investigation, Project administration, Writing – review & editing. JW: Data curation, Investigation, Project administration, Writing – review & editing. X-yS: Data curation, Investigation, Writing – review & editing. J-lC: Investigation, Project administration, Writing – review & editing. C-xW: Investigation, Project administration, Writing – review & editing. W-cJ: Conceptualization, Formal analysis, Methodology, Project administration, Resources, Writing – review & editing. XL: Conceptualization, Formal analysis, Funding acquisition, Methodology, Resources, Writing – review & editing.

## Footnotes

^1^ http://www.acuprime.com/

^2^ https://www.R-project.org/

## References

[1] Barbarot S Auziere S Gadkari A Girolomoni G Puig L Simpson EL. Epidemiology of atopic dermatitis in adults: results from an international survey. Allergy. (2018) 73:1284–93. doi: 10.1111/all.13401 29319189

[2] Kang MC Cho K Lee JH Subedi L Yumnam S Kim SY. Effect of resveratrol-enriched rice on skin inflammation and pruritus in the NC/NGA mouse model of atopic dermatitis. Int J Mol Sci. (2019) 20:1428. doi: 10.3390/ijms20061428 30901835 PMC6471349

[3] Johansson SG Bieber T Dahl R Friedmann PS Lanier BQ Lockey RF. Revised nomenclature for allergy for global use: report of the nomenclature review committee of the World Allergy Organization, October 2003. J Allergy Clin Immunol. (2004) 113:832–6. doi: 10.1016/j.jaci.2003.12.591, PMID: 15131563

[4] Zhao B. Chinese clinical dermatology, Phoenix science press. vol. 1. 1st ed (2009). 12 p.

[5] Raveendran R. Tips and tricks for controlling eczema. Immunol Allergy Clin N Am. (2019) 39:521–33. doi: 10.1016/j.iac.2019.07.006, PMID: 31563186

[6] Norén P Hagströmer L Alimohammadi M Melin L. The positive effects of habit reversal treatment of scratching in children with atopic dermatitis: a randomized controlled study. Br J Dermatol. (2018) 178:665–73. doi: 10.1111/bjd.16009, PMID: 28940213

[7] Melin L Frederiksen T Noren P Swebilius BG. Behavioural treatment of scratching in patients with atopic dermatitis. Br J Dermatol. (1986) 115:467–74. doi: 10.1111/j.1365-2133.1986.tb06241.x 3778815

[8] Bender BG Ballard R Canono B Murphy JR Leung DY. Disease severity, scratching, and sleep quality in patients with atopic dermatitis. J Am Acad Dermatol. (2008) 58:415–20. doi: 10.1016/j.jaad.2007.10.010, PMID: 18280338

[9] Bao Q Chen L Lu Z Ma Y Guo L Zhang S. Association between eczema and risk of depression: a systematic review and meta-analysis of 188,495 participants. J Affect Disord. (2018) 238:458–64. doi: 10.1016/j.jad.2018.05.007, PMID: 29929155

[10] Pereira MP Kremer AE Mettang T Ständer S. Chronic pruritus in the absence of skin disease: pathophysiology, diagnosis and treatment. Am J Clin Dermatol. (2016) 17:337–48. doi: 10.1007/s40257-016-0198-0, PMID: 27216284

[11] Hajar T Leshem YA Hanifin JM Nedorost ST Lio PA Paller AS. A systematic review of topical corticosteroid withdrawal ("steroid addiction") in patients with atopic dermatitis and other dermatoses. J Am Acad Dermatol. (2015) 72:541–549.e2. doi: 10.1016/j.jaad.2014.11.024, PMID: 25592622

[12] Aubert-Wastiaux H Moret L le Rhun A Fontenoy AM Nguyen JM Leux C. Topical corticosteroid phobia in atopic dermatitis: a study of its nature, origins and frequency. Br J Dermatol. (2011) 165:808–14. doi: 10.1111/j.1365-2133.2011.10449.x, PMID: 21671892

[13] Li AW Yin ES Antaya RJ. Topical corticosteroid phobia in atopic dermatitis: a systematic review. JAMA Dermatol. (2017) 153:1036–42. doi: 10.1001/jamadermatol.2017.2437 28724128

[14] Sidbury R Davis DM Cohen DE Cordoro KM Berger TG Bergman JN. Guidelines of care for the management of atopic dermatitis: section 3. Management and treatment with phototherapy and systemic agents. J Am Acad Dermatol. (2014) 71:327–49. doi: 10.1016/j.jaad.2014.03.030, PMID: 24813298 PMC4410179

[15] Weisshaar E Szepietowski JC Darsow U Misery L Wallengren J Mettang T. European guideline on chronic pruritus. Acta Derm Venereol. (2012) 92:563–81. doi: 10.2340/00015555-1400 22790094

[16] He A Feldman SR Fleischer AB Jr. An assessment of the use of antihistamines in the management of atopic dermatitis. J Am Acad Dermatol. (2018) 79:92–6. doi: 10.1016/j.jaad.2017.12.077 29317281

[17] Misery L Belloni Fortina A el Hachem M Chernyshov P von Kobyletzki L Heratizadeh A. A position paper on the management of itch and pain in atopic dermatitis from the International Society of Atopic Dermatitis (ISAD)/oriented patient-education network in dermatology (OPENED) task force. J Eur Acad Dermatol Venereol. (2021) 35:787–96. doi: 10.1111/jdv.16916, PMID: 33090558

[18] Du Buske LM. Clinical comparison of histamine H1-receptor antagonist drugs. J Allergy Clin Immunol. (1996) 98:S307–18. doi: 10.1016/s0091-6749(96)80116-3 8977542

[19] Brown S Reynolds NJ. Atopic and non-atopic eczema. BMJ. (2006) 332:584–8. doi: 10.1136/bmj.332.7541.584, PMID: 16528081 PMC1397720

[20] Jwo JY Chiou K Tsai J Huang YC Lin CY. Efficacy of acupuncture for treatment of atopic eczema and chronic eczema: a systematic review and meta-analysis. Acta Derm Venereol. (2022) 102:adv00791. doi: 10.2340/actadv.v102.4380, PMID: 36200506 PMC9677264

[21] Xing M Yan X Yang S Li L Gong L Liu H. Effects of moving cupping therapy for plaque psoriasis: study protocol for a randomized multicenter clinical trial. Trials. (2020) 21:229. doi: 10.1186/s13063-020-4155-0, PMID: 32102679 PMC7045603

[22] Hon KL Chan BC Leung PC. Chinese herbal medicine research in eczema treatment. Chin Med. (2011) 6:17. doi: 10.1186/1749-8546-6-17, PMID: 21527032 PMC3110124

[23] Salameh F Perla D Solomon M Gamus D Barzilai A Greenberger S. The effectiveness of combined Chinese herbal medicine and acupuncture in the treatment of atopic dermatitis. J Altern Complement Med. (2008) 14:1043–8. doi: 10.1089/acm.2008.0162, PMID: 18990051

[24] Hwang J Lio PA. Acupuncture in dermatology: an update to a systematic review. J Altern Complement Med. (2021) 27:12–23. doi: 10.1089/acm.2020.0230, PMID: 32955916

[25] Huang J Li G Xiang J Yin D Chi R. Immunohistochemical study of serotonin in lesions of chronic eczema. Int J Dermatol. (2004) 43:723–6. doi: 10.1111/j.1365-4632.2004.02196.x, PMID: 15485527

[26] Park HJ Ahn S Lee H Hahm DH Kim K Yeom M. Acupuncture ameliorates not only atopic dermatitis-like skin inflammation but also acute and chronic serotonergic itch possibly through blockade of 5-HT(2) and 5-HT(7) receptors in mice. Brain Behav Immun. (2021) 93:399–408. doi: 10.1016/j.bbi.2021.01.027 33524554

[27] Kesting MR Thurmüller P Hölzle F Wolff KD Holland-Letz T Stücker M. Electrical ear acupuncture reduces histamine-induced itch (alloknesis). Acta Derm Venereol. (2006) 86:399–403. doi: 10.2340/00015555-0115, PMID: 16955182

[28] Lundeberg T Bondesson L Thomas M. Effect of acupuncture on experimentally induced itch. Br J Dermatol. (1987) 117:771–7. doi: 10.1111/j.1365-2133.1987.tb07359.x, PMID: 3426954

[29] Back SK Jeong KY Li C Lee J Lee SB Na HS. Chronically relapsing pruritic dermatitis in the rats treated as neonate with capsaicin; a potential rat model of human atopic dermatitis. J Dermatol Sci. (2012) 67:111–9. doi: 10.1016/j.jdermsci.2012.05.006, PMID: 22721998

[30] Jung DL Lee SD Choi IH Na HS Hong SU. Effects of electroacupuncture on capsaicin-induced model of atopic dermatitis in rats. J Dermatol Sci. (2014) 74:23–30. doi: 10.1016/j.jdermsci.2013.11.015, PMID: 24418195

[31] Liu S Wang Z Su Y Qi L Yang W Fu M. A neuroanatomical basis for electroacupuncture to drive the vagal-adrenal axis. Nature. (2021) 598:641–5. doi: 10.1038/s41586-021-04001-4, PMID: 34646018 PMC9178665

[32] Pfab F Huss-Marp J Gatti A Fuqin J Athanasiadis GI Irnich D. Influence of acupuncture on type I hypersensitivity itch and the wheal and flare response in adults with atopic eczema - a blinded, randomized, placebo-controlled, crossover trial. Allergy. (2010) 65:903–10. doi: 10.1111/j.1398-9995.2009.02284.x, PMID: 20002660

[33] Lx Z. A study on the central response characteristics of acupuncture in the treatment of chronic spontaneous urticaria. Chengdu City, Sichuan Province, China: University of Traditional Chinese Medicine (2021).

[34] Yang Q Liu M Li X Zheng J. The benefit of a ceramide-linoleic acid-containing moisturizer as an adjunctive therapy for a set of xerotic dermatoses. Dermatol Ther. (2019) 32:e13017. doi: 10.1111/dth.13017, PMID: 31276265

[35] Reich A Heisig M Phan NQ Taneda K Takamori K Takeuchi S. Visual analogue scale: evaluation of the instrument for the assessment of pruritus. Acta Derm Venereol. (2012) 92:497–501. doi: 10.2340/00015555-1265, PMID: 22102095

[36] Hanifin JM Thurston M Omoto M Cherill R Tofte SJ Graeber M. The eczema area and severity index (EASI): assessment of reliability in atopic dermatitis. EASI evaluator group. Exp Dermatol. (2001) 10:11–8. doi: 10.1034/j.1600-0625.2001.100102.x 11168575

[37] Finlay AY Khan GK. Dermatology life quality index (DLQI)--a simple practical measure for routine clinical use. Clin Exp Dermatol. (1994) 19:210–6. doi: 10.1111/j.1365-2230.1994.tb01167.x 8033378

[38] Wu CY Huang HY Pan WC Liao SL Hua MC Tsai MH. Allergic diseases attributable to atopy in a population sample of Asian children. Sci Rep. (2021) 11:16052. doi: 10.1038/s41598-021-95579-2, PMID: 34362983 PMC8346539

[39] Avena-Woods C. Overview of atopic dermatitis. Am J Manag Care. (2017) 23:S115–s123. PMID: 28978208

[40] Weidinger S Novak N. Atopic dermatitis. Lancet. (2016) 387:1109–22. doi: 10.1016/s0140-6736(15)00149-x 26377142

[41] Wallach D Taïeb A. Atopic dermatitis/atopic eczema. Chem Immunol Allergy. (2014) 100:81–96. doi: 10.1159/000358606 24925387

[42] Sawangjit R Dilokthornsakul P Lloyd-Lavery A Lai NM Dellavalle R Chaiyakunapruk N. Systemic treatments for eczema: a network meta-analysis. Cochrane Database Syst Rev. (2020) 9:Cd013206. doi: 10.1002/14651858.CD013206.pub2 32927498 PMC8128359

[43] David Boothe W Tarbox JA Tarbox MB. Atopic dermatitis: pathophysiology. Adv Exp Med Biol. (2017) 1027:21–37. doi: 10.1007/978-3-319-64804-0_3 29063428

[44] Zeng Z Li M Zeng Y Zhang J Zhao Y Lin Y. Potential acupoint prescriptions and outcome reporting for acupuncture in atopic eczema: a scoping review. Evid Based Complement Alternat Med. (2021) 2021:9994824–8. doi: 10.1155/2021/9994824, PMID: 34257697 PMC8257338

[45] Sun X Zhou X Wei Y Yang W Huang N Ding Y. Our choice: study protocol for a randomized controlled trial for optimal implementation of psoriasis treatment by the integration of Chinese and western medicine. Trials. (2020) 21:299. doi: 10.1186/s13063-020-4209-3, PMID: 32228720 PMC7106809

[46] Ru Y Yan XN Yang SQ Gong LP Li LE Chen J. Oral Taodan granules for mild-to-moderate psoriasis vulgaris: protocol for a randomized, double-blind, multicenter clinical trial. Ann Transl Med. (2019) 7:488. doi: 10.21037/atm.2019.09.05, PMID: 31700924 PMC6803197

[47] Kang S Kim YK Yeom M Lee H Jang H Park HJ. Acupuncture improves symptoms in patients with mild-to-moderate atopic dermatitis: a randomized, sham-controlled preliminary trial. Complement Ther Med. (2018) 41:90–8. doi: 10.1016/j.ctim.2018.08.013, PMID: 30477869

[48] Ernst E. The usage of complementary therapies by dermatological patients: a systematic review. Br J Dermatol. (2000) 142:857–61. doi: 10.1046/j.1365-2133.2000.03463.x, PMID: 10809840

[49] Vieira BL Lim NR Lohman ME Lio PA. Complementary and alternative medicine for atopic dermatitis: an evidence-based review. Am J Clin Dermatol. (2016) 17:557–81. doi: 10.1007/s40257-016-0209-1, PMID: 27388911

[50] Zhao L Liu L Xu X Qu Z Zhu Y Li Z. Electroacupuncture inhibits hyperalgesia by alleviating inflammatory factors in a rat model of migraine. J Pain Res. (2020) 13:75–86. doi: 10.2147/jpr.S225431, PMID: 32021397 PMC6968809

[51] Park MB Ko E Ahn C Choi H Rho S Shin MK. Suppression of IgE production and modulation of Th1/Th2 cell response by electroacupuncture in DNP-KLH immunized mice. J Neuroimmunol. (2004) 151:40–4. doi: 10.1016/j.jneuroim.2004.02.003, PMID: 15145602

[52] Carlsson CP Wallengren J. Therapeutic and experimental therapeutic studies on acupuncture and itch: review of the literature. J Eur Acad Dermatol Venereol. (2010) 24:1013–6. doi: 10.1111/j.1468-3083.2010.03585.x, PMID: 20337812

